# Molecular Imaging-Derived Biomarker of Cardiac Nerve Integrity — Introducing High NET Affinity PET Probe ^18^F-AF78

**DOI:** 10.7150/thno.63205

**Published:** 2022-05-24

**Authors:** Xinyu Chen, Rudolf A. Werner, Kazuhiro Koshino, Naoko Nose, Saskia Mühlig, Steven P Rowe, Martin G. Pomper, Constantin Lapa, Michael Decker, Takahiro Higuchi

**Affiliations:** 1 Nuclear Medicine, Faculty of Medicine, University of Augsburg, Augsburg, Germany.; 2 Department of Nuclear Medicine and Comprehensive Heart Failure Center, University Hospital of Würzburg, Würzburg, Germany.; 3 Division of Nuclear Medicine, The Russell H Morgan Department of Radiology and Radiological Science, Johns Hopkins University School of Medicine, Baltimore, MD, U.S.; 4 Department of Systems and Informatics, Hokkaido Information University, Ebetsu, Hokkaido, Japan.; 5 Faculty of Medicine, Dentistry and Pharmaceutical Sciences, Okayama University, Okayama, Japan.; 6 Institute of Pharmacy and Food Chemistry, University of Würzburg, Würzburg, Germany.

**Keywords:** norepinephrine transporter, sympathetic nervous system, cardiac innervation imaging, radiotracer kinetics, nonhuman primates, T-shaped π-π stacking

## Abstract

**Background:** Radiolabeled agents that are substrates for the norepinephrine transporter (NET) can be used to quantify cardiac sympathetic nervous conditions and have been demonstrated to identify high-risk congestive heart failure (HF) patients prone to arrhythmic events. We aimed to fully characterize the kinetic profile of the novel ^18^F-labeled NET probe AF78 for PET imaging of the cardiac sympathetic nervous system (SNS) among various species.

**Methods:** ^18^F-AF78 was compared to norepinephrine (NE) and established SNS radiotracers by employing *in vitro* cell assays, followed by an *in vivo PET imaging* approach with healthy rats, rabbits and nonhuman primates (NHPs). Additionally, chase protocols were performed in NHPs with NET inhibitor desipramine (DMI) and the NE releasing stimulator tyramine (TYR) to investigate retention kinetics in cardiac SNS.

**Results:** Relative to other SNS radiotracers, ^18^F-AF78 showed higher transport affinity via NET in a cell-based competitive uptake assay (IC_50_ 0.42 ± 0.14 µM), almost identical to that of NE (IC_50_, 0.50 ± 0.16 µM, n.s.). In rabbits and NHPs, initial cardiac uptake was significantly reduced by NET inhibition. Furthermore, cardiac tracer retention was not affected by a DMI chase protocol but was markedly reduced by intermittent TYR chase, thereby suggesting that ^18^F-AF78 is stored and can be released via the synaptic vesicular turnover process. Computational modeling hypothesized the formation of a T-shaped π-π stacking at the binding site, suggesting a rationale for the high affinity of ^18^F-AF78.

**Conclusion:** ^18^F-AF78 demonstrated high *in vitro* NET affinity and advantageous *in vivo* radiotracer kinetics across various species, indicating that ^18^F-AF78 is an SNS imaging agent with strong potential to guide specific interventions in cardiovascular medicine.

## Introduction

Various clinical conditions, such as heart failure (HF) 1, 2, diabetes-associated cardiac autonomic neuropathy (CAN) 3, dilated or hypertrophic cardiomyopathy 4, 5, aortic stenosis 6 or infarcted myocardium, are associated with alterations of the sympathetic nervous system (SNS) 7. Serving as the backbone of neurohumoral radionuclide imaging, cardiac SNS radiotracers using either single-photon emission computed tomography (SPECT) or positron emission tomography (PET) are mostly structurally related to physiological norepinephrine (NE) 8, 9. Neurohumoral cardiac radiotracers share similar pathways with NE and are transported into neuronal cells via the NE transporter (NET), and some of these radiotracers are subsequently stored in presynaptic vesicles.

Such radiotracers have been suggested to provide a noninvasive read-out indicating the level of denervated myocardium 10. The radiotracer ^123^I-*meta*-iodobenzylguanidine (^123^I-MIBG) was the first and only approved molecular imaging agent by the U.S. Food and Drug Administration for the scintigraphic assessment of myocardial sympathetic innervation. In contrast to such a global myocardial assessment, the increased spatiotemporal resolution of PET technology allows for a more precise read-out of regional neuronal denervation, e.g., the amount of denervated myocardium in the border zone after myocardial infarction relative to remote myocardium 11.

Substantial evidence has been obtained on neurohumoral image-guided strategies in various clinical scenarios, including implantable cardiac device (ICD) insertion or prognostication 8, 12, 13. For instance, the “AdreView Myocardial Imaging for Risk Evaluation in Heart Failure (ADMIRE-HF)” trial demonstrated the superior prognostic value of ^123^I-MIBG scintigraphy for major cardiovascular events in ischemic and nonischemic HF patients compared to that of other established parameters 14. Based on these encouraging results, the PAREPET trial linked denervated myocardium quantified by ^11^C-*meta*-hydroxyephedrine (^11^C-HED) PET to an increased risk for sudden cardiac death in ischemic cardiomyopathy patients, independent of left ventricular ejection fraction and infarct volume 15. The use of ^18^F-labeled SNS PET radiotracers has also increased recently; these tracers have the advantage of a significantly longer half-life (110 min vs. 20 min for carbon-11), allowing the dispatch from central cyclotron facilities for improved cost-effectiveness or implementation of additional delayed image protocols. Thirty years ago, ^18^F-6-fluorodopamine (^18^F-6F-DA) was introduced as the first ^18^F-labeled NET radiotracer 16, demonstrating its usefulness in evaluating various scenarios, such as neuroendocrine tumors 17, cardiac SNS changes 18 and sympathetic denervation in Parkinson's disease (PD) 19. Recently, the novel ^18^F-labeled radiotracer AF78 was introduced as a NET-targeting probe with a phenethylguanidine core structure. AF78 is characterized by an easy radiolabeling procedure and high radiolabeling yield, allowing for high throughput even in a busy PET practice 20. However, prior to routine human use, a precise understanding of the catecholamine radiotracer handling of ^18^F-AF78 at the nerve terminal is indispensable. Such accurate characterization of ^18^F-AF78 at the nerve terminal may pave the way for image-guided molecular strategies in various clinical scenarios, including prediction of arrhythmias, guiding resynchronization therapies, or monitoring complex cardiac interventions, such as valve replacement. In the present study, we aimed to fully decipher the kinetic profile of ^18^F-AF78 of the cardiac SNS by employing *in vitro* cell assays and high-resolution PET imaging among various species.

## Methods

Experimental protocols were approved by the Animal Ethics Committee of the National Cerebral and Cardiovascular Center, Research Institute, Osaka, Japan (Approval number 18019) and conducted in strict accordance with the *Guide for the Care and Use of Laboratory Animals,* published by the U.S. National Institute of Health 21, and the ARRIVE guidelines.

### Radiolabeling

**Figure 1:**
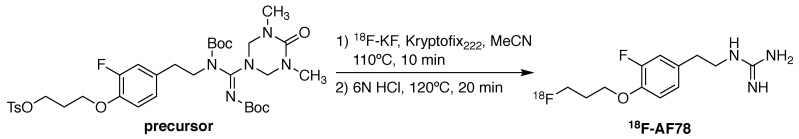
Radiolabeling of ^18^F-AF78 with a one-pot two-step labeling procedure from a precursor with fully protected guanidine. Kryptofix_222_ = 4,7,13,16,21,24-Hexaoxa-1,10-diazabicyclo[8.8.8]hexacosane, MeCN = acetonitrile, HCl = hydrochloride.

^18^F-AF78 was synthesized as described previously 20. In short, a solution of the precursor (4.8 mg, 0.066 mmol) and Kryptofix_222_ (22.5 mg, 0.060 mmol) in dry acetonitrile (3 mL) was added to the isolated and dried ^18^F-KF. The mixture was heated at 110 °C for 10 min, followed by the addition of hydrochloric acid (6 N, 0.3 mL) and further heating at 120 °C for 20 min (**Figure 1**). After quenching the reaction, the mixture was purified via semipreparative high-performance liquid chromatography. The collected fraction was diluted with water (10 mL) and trapped on a preconditioned Sep-Pak C18 cartridge, which was then washed with water (5 mL). ^18^F-AF78 was eluted with ethanol (1 mL). After removing the solvent with nitrogen flow, the target tracer was diluted with saline to the required concentration for further application. The radiochemical purity of ^18^F-AF78 was consistently > 97%, with molar activity > 56 GBq/µmol.

### Cell culture and competitive uptake assay

SK-N-SH (human neuroblastoma cell line) cells naturally expressing NET were cultivated according to the instructions from the supplier (Sigma-Aldrich Chemie GmbH, Taufkirchen, Germany). The cells were seeded one day before the competitive assay into a 24-well plate at 1 × 10^5^ cells/well and incubated overnight at 37 °C. In the presence of 100 µM pargyline (monoamine oxidase inhibitor) and 20 µM pyrogallol (catechol-O-methyltransferase inhibitor), ^3^H-NE (PerkinElmer, Germany) in 0.1% BSA/DMEM (12 kBq/mL) was added to each well together with various concentrations of either NET selective inhibitor desipramine (DMI) or testing compounds, including NE as reference, ranging from a final concentration of 10^-10^ to 10^-3^ M. The plate was incubated at 37 °C for 60 min. The supernatant was removed, and the cells were washed with ice-cold phosphate-buffered saline buffer (2 × 1 ml) followed by the addition of NaOH solution (0.1 N, 500 μl). Cell lysates were collected and measured in a liquid scintillation analyzer 10. Nonspecific uptake was determined by incubating at 4 °C, which was negligible in all cases. The cold references of 1-(3-bromo-4-(^18^F-3-fluoropropoxy)benzyl)guanidine/flubrobenguane (FBBG), 4-fluoro-3-hydroxyphenethylguanidine (4F-MHPG) and 3-fluoro-4-hydroxyphenethylguanidine (3F-PHPG) were synthesized as described with final purity ≥ 95% on HPLC, and were structures confirmed by NMR in accordance with the literature 22, 23. The other reference compounds are commercially available with purity ≥ 95% and were as follows: norepinephrine bitartrate and phenoxybenzamine (PhB) hydrochloride (TCI Europe, Zwijndrecht, Belgium), desipramine (DMI) hydrochloride (Sigma-Aldrich Chemie GmbH, Taufkirchen, Germany), 6-fluorodopamine and *meta*-hydroxylephedrine (HED) hydrochloride (ABX advanced biochemical compounds GmbH, Radeberg, Germany), nonradioactive *meta*-iodobenzylguanidine (MIBG) hydrochloride (Activate Scientific, Prien, Germany), and ^123^I-MIBG (FUJIFILM Toyama Chemical Co., Ltd., Chiba, Japan).

### Animal preparation

Male Wistar rats (*n* = 4, weighing 250-400 g) and New Zealand white rabbits (*n* = 5, weighing 3.2-3.6 kg) were used for cardiac uptake studies. Anesthesia was induced and maintained during the experiment by 2% isoflurane 24. Large animal PET studies were performed on cynomolgus monkeys (*n* = 4, weighing 3.9-4.2 kg). Induction of anesthesia in NHPs was conducted by intramuscular injection of ketamine (1.5 mg/kg) and xylazine (0.6 mg/kg) to allow the preparation and handling of the animals. After a tracheal cannula was inserted, 1.5% sevoflurane (SEVOFLURANE Inhalation Solution, Pfizer Japan Inc., Tokyo, Japan) vaporized with 100% oxygen was inhaled, and the tidal volume and respiratory rate of the ventilator were monitored and kept in the normal range throughout the imaging sessions with an anesthesia workstation (Apollo®, Drägerwerk AG & Co. KGaA, Lübeck, Germany) 25.

### PET imaging, biodistribution and kinetic studies

A small-animal PET system (microPET FOCUS 120, Siemens, Erlangen, Germany) was used for the rat studies. A 30-min dynamic scan was initiated immediately after the radiotracer injection (10-20 MBq) via the tail vein (in a bolus). For the blocking study in rats, PhB (50 mg/kg, *n* = 4) was injected 10 min before radiotracer administration. The list-mode data were sorted into three-dimensional reconstructions, which were then rebinned with full three-dimensional binning to reconstruct dynamic images. Data were collected in the following frames: 10 s × 12, 30 s × 6, and 300 s × 5. For the biodistribution studies, 10 min after radiotracer injection (1-2 MBq) under anesthesia, the animals were euthanized for the control or PhB blocking group (50 mg/kg, *n* = 4-6). Another group of animals was euthanized 60 min after radiotracer administration (1-2 MBq, *n* = 4-6) to investigate late-phase uptake. Organs of interest were harvested for tissue counting with a γ-counter. The organ-to-blood ratios were calculated following weight and decay correction of tissue counts.

^18^F-AF78 (3-4 MBq) and ^123^I-MIBG (approximately 2.5 MBq) were injected into rabbits maintained under anesthesia via the marginal ear vein in a single dose. The NET blocker DMI (1.5 mg/kg) was injected 10 min before radiotracer administration in the blocking study. A 10-min dynamic scan was initiated immediately after the radiotracer injection. The animals were euthanized immediately after the scan, and the organs of interest were harvested and measured in a gamma counter to calculate biodistribution.

NHPs were maintained under anesthesia during imaging and were studied with a PET scanner (PCA-2000A, Toshiba Medical Systems Corporation, Tochigi, Japan). After a 5-min transmission scan and a bolus intravenous (i.v.) injection of the radiotracer ^18^F-AF78 (approximately 20 MBq) via the saphenous vein, a 60-min dynamic PET scan (10 s × 12, 30 s × 6, and 300 s × 11) was initiated. For the blocking study, DMI was injected 10 min before radiotracer administration, after which the PET scan commenced immediately (*n* = 4). For the DMI chase study, DMI (1 mg/kg, *n* = 4) was injected 10 min after radiotracer administration, while the PET scan was initiated simultaneously with radiotracer administration. The catecholamine releasing stimulator tyramine (TYR) was also used in a chase protocol, with 50 µg/kg each injection (*n* = 4) injected intermittently (5 times, 10-min intervals) after radiotracer administration. The radiotracers ^18^F-AF78 and ^123^I-MIBG were formulated in saline for the animal studies. Control animals received normal saline. The inhibitors PhB, DMI and TYR were first dissolved in a minimum amount of DMSO and diluted with saline for administration with a final concentration of DMSO lower than 10%. The experimental timeline is provided in the supplementary material.

PET imaging for rats, rabbits and NHPs was performed from the chest to the upper abdomen, including the heart, lungs, liver and part of the intestines. The reconstructed images were analyzed using AMIDE imaging software (version 1.0.1). For kinetic studies in rats and NHPs, the tissue uptake was determined as standardized uptake values (SUVs), from which time-activity curves of different organs, e.g., heart, blood, lung and liver, were generated with transmission computed tomography images for topographic localization.

### Statistics

All results are displayed as the mean ± SD. A two-tailed paired Student's *t test* was used to compare differences between two dependent groups, and a two-tailed independent Student's *t test* was used to compare differences between independent groups. Multiple group comparisons were performed using analysis of variance (two-way ANOVA). A *P* value of less than 0.05 was assumed to be statistically significant. Statistical analysis was performed with GraphPad Prism (version 8.4.3 GraphPad Software, San Diego, USA).

## Results

**The affinity of AF78 to NET is similar to that of NE and is higher to that of established SNS radiotracers.** AF78 showed almost identical transporting affinity by NET when compared to the physiological reference NE (IC_50_ values 0.42 ± 0.14 µM vs. 0.50 ± 0.16 µM, respectively, n.s.). When compared to other established ^18^F-labeled SNS radiotracers, the cell uptake affinity of AF78 by NET was significantly higher than that of 4F-MHPG (IC_50_, 2.78 ± 1.22 µM, *P* ≤ 0.01) and FBBG (IC_50_, 2.28 ± 1.05 µM, *P* ≤ 0.05) but not 3F-PHPG (IC_50_, 0.78 ± 0.060 µM, n.s.). In addition, a 4.2-fold higher affinity of AF78 relative to MIBG was noted (IC_50_, 1.86 ± 0.30 µM, *P* ≤ 0.05). HED revealed an IC_50_ of 0.39 ± 0.11 µM, (n.s.), which is comparable to that of AF78. DMI, a selective and specific inhibitor of NET, was used as a positive control (**Table 1**).

**Figure 2:**
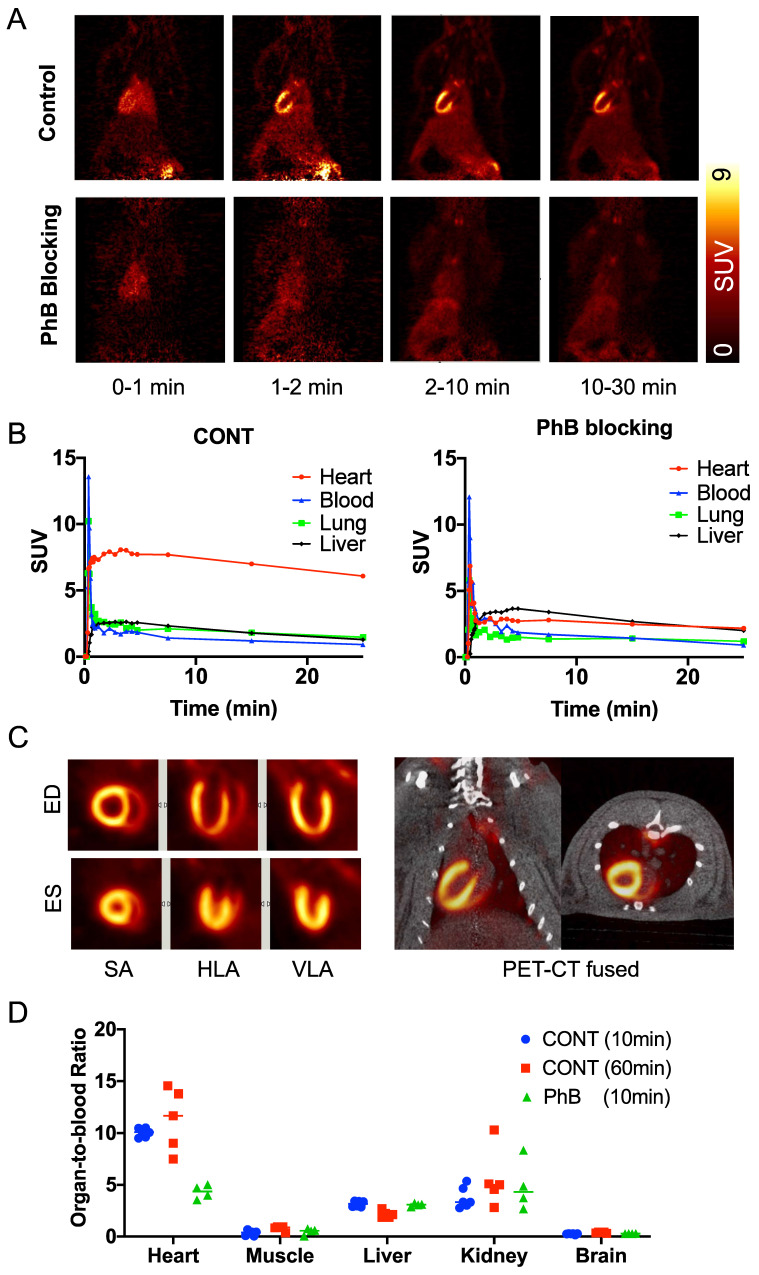
**(A)** Representative dynamic imaging of rat hearts (coronal axis) with or without norepinephrine transporter blocking by using nonselective inhibitor phenoxybenzamine (PhB, 10 min after tracer iv), demonstrating stable delineation of the myocardium over all imaging time-points in controls but not in PhB pretreated animals. **(B)** Time-activity curves, expressed as standardized uptake values (SUVs), were generated from dynamic positron emission tomography images in rats and after blockade with PhB. Quantitative values in the myocardium remained stable in the heart over all time points but were significantly reduced in PhB pretreated animals. Activity in the liver was not affected by pretreatment with PhB. (**C**) ECG-gated cardiac ^18^F-AF78 PET images (20-30 min) at endo diastolic phase (ED) and endo systolic phase (ES) in the short axis (SA), horizontal long-axis (HLA) and vertical long-axis (VLA) (left). Coronal fused images from PET-CT scans (right). (**D**) Organ-to-blood ratios calculated from the biodistribution study 10 (blue) and 60 (red) min after tracer administration in the control group, along with pretreatment with PhB (green). *n* = 4-6 animals per group were investigated.

**Biodistribution of ^18^F-AF78 allows for homogenous delineation of the heart in rats.** Homogeneous ^18^F-AF78 distribution throughout the left ventricle (LV) could be visualized in healthy Wistar rats. Administration of the radiotracer after PhB pretreatment demonstrated a focal decrease in radiotracer accumulation that remained stable throughout the imaging protocol (**Figure 2A**). Time-activity curves showed sustained intense radiotracer uptake in the heart but continuous low values for both the blood pool and liver in control rats (**Figure 2B**). Upon PhB pretreatment, cardiac uptake decreased rapidly after the initial perfusion, reaching comparable values to that of the blood pool. Stable cardiac uptake was observed in the biodistribution study showing unchanged the organ-to-blood ratios in the early (10 min) and late (60 min) phases. In the blocking study using the nonselective NET blocker PhB, only uptake in the heart, not in any other organ, was reduced, demonstrating NET-mediated specific radiotracer uptake (**Figure 2D**).

**Figure 3:**
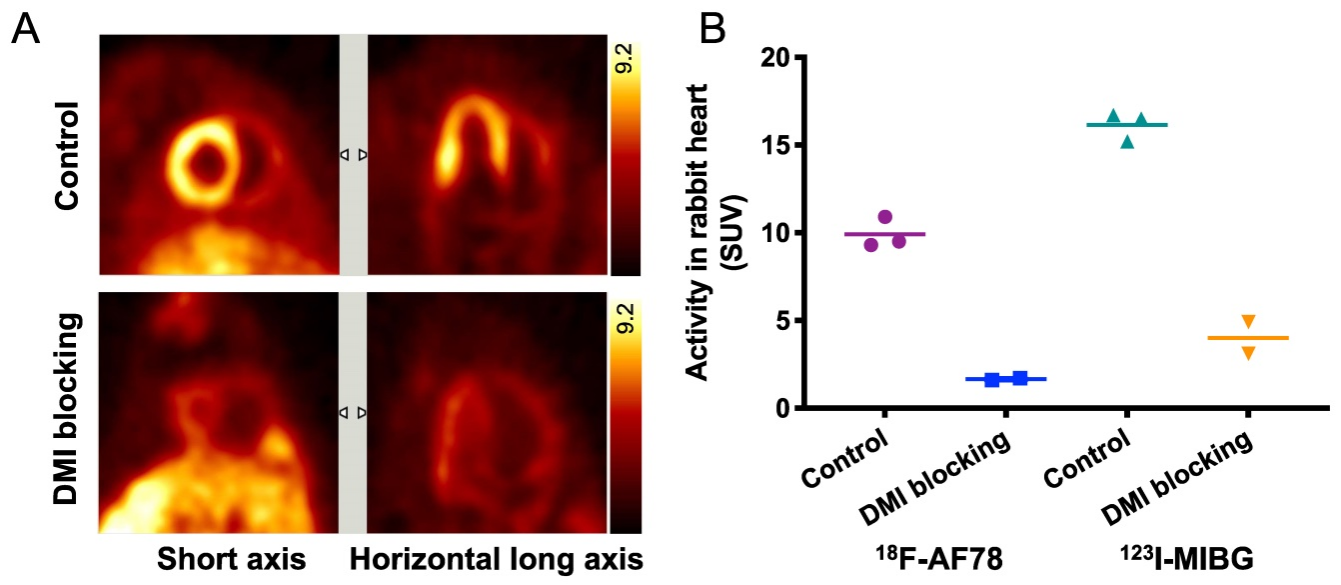
** (A)** PET imaging of ^18^F-AF78 in rabbit hearts in the control and NET blocking conditions, in which the specificity of NET-mediated tracer uptake has been proven by the greatly decreased cardiac uptake after pretreatment with the NET-selective inhibitor desipramine (DMI). **(B)** Activity in the heart after the administration of ^18^F-AF78 and ^123^I-MIBG in rabbits presented as standardized uptake values (SUV) in both control and NET blocking conditions. Cardiac uptake of both radiotracers was reduced significantly in the DMI pretreatment group, demonstrating comparable *in vivo* features. *n* = 5 animals were investigated.

**Specific cardiac uptake of ^18^F-AF78 in rabbits showed comparable features to ^123^I-MIBG.** Distinct cardiac imaging showed a homogeneous distribution of ^18^F-AF78 throughout the LV in healthy rabbits, while this uptake diminished after pretreatment with the NET blocker DMI (**Figure 3A**). By comparing ^18^F-AF78 and ^123^I-MIBG in the same animals, although the uptake of the former was lower than that of the latter in the controls, the mean SUV after DMI blockade — which can be considered background — was lower, demonstrating comparable contrast with and a better signal-to-background ratio than obtained with ^123^I-MIBG (**Figure 3B**).

**Figure 4:**
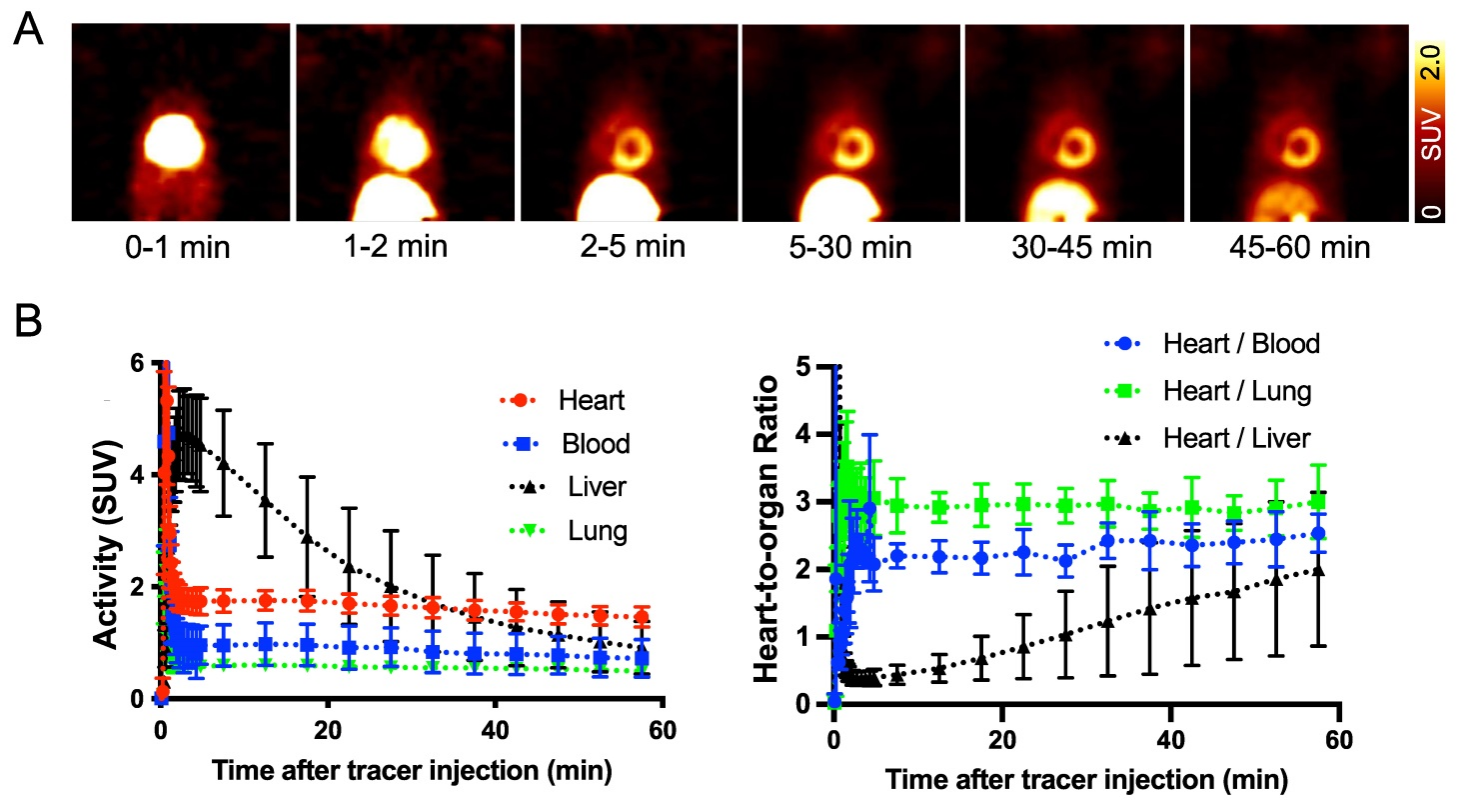
**(A)** Dynamic positron emission tomography images of ^18^F-AF78 divided by different time frames in nonhuman primates, demonstrating stable delineation of the radiotracer in the myocardium with declining radiotracer uptake in the liver over time. Little or no retention of the tracer in the lungs was observed over time, providing good heart-to-lung contrast. **(B)** Time-activity curves (left) of ^18^F-AF78 in the myocardium, blood, liver, and lung presented as standardized uptake values (SUVs), with stable SUVs in the heart but a decline in the liver over time. A plot of uptake ratios *versus* time in minutes (right) demonstrated stable heart/blood and heart/lung ratios in comparison to increased heart/liver ratios due to hepatic tracer washout. *n* = 4 animals were investigated.

**^18^F-AF78 shows an positive biodistribution in NHPs, revealing high-contrast SNS imaging at late phases.** In healthy NHPs, radiotracer accumulation with clear delineation of the LV could already be visualized 2-5 min postinjection. Almost no uptake could be identified in the blood pool, whereas uptake in the liver decreased slowly over time. Therefore, high-contrast images with reduced hepatic uptake were obtained 45-60 min postinjection, supporting the notion of improved image quality at later phases. Little or no radiotracer retention in the lungs was observed over time (**Figure 4A**). Time-activity curves derived from ^18^F-AF78 dynamic images in controls showed high cardiac radiotracer activity after the initial blood pool washout that plateaued throughout the entire scan time with heart-to-blood pool ratios of 2.03 ± 0.53 at 5 min and 1.82 ± 0.71 at 60 min (**Figure 4B**). Relative to cardiac uptake, radiotracer accumulation in the liver was elevated at 5 min postinjection (heart-to-liver ratio at 5 min, 0.38 ± 0.29) but decreased over time (heart-to-liver ratios at 30 min, 0.95 ± 0.20 and 60 min, 1.60 ± 0.38, respectively). Further corroborating the improved image quality at later phases, the absolute SUVs of ^18^F-AF78 in the blood pool (0.72 ± 0.34) and liver (0.91 ± 0.47) were almost identical at 60 min postinjection, whereas cardiac uptake was higher (1.46 ± 0.18). The heart/liver ratio neared the same level as the heart/blood ratio 60 min after tracer administration, resulting in high-contrast cardiac imaging (**Figure 4B**).

**Figure 5:**
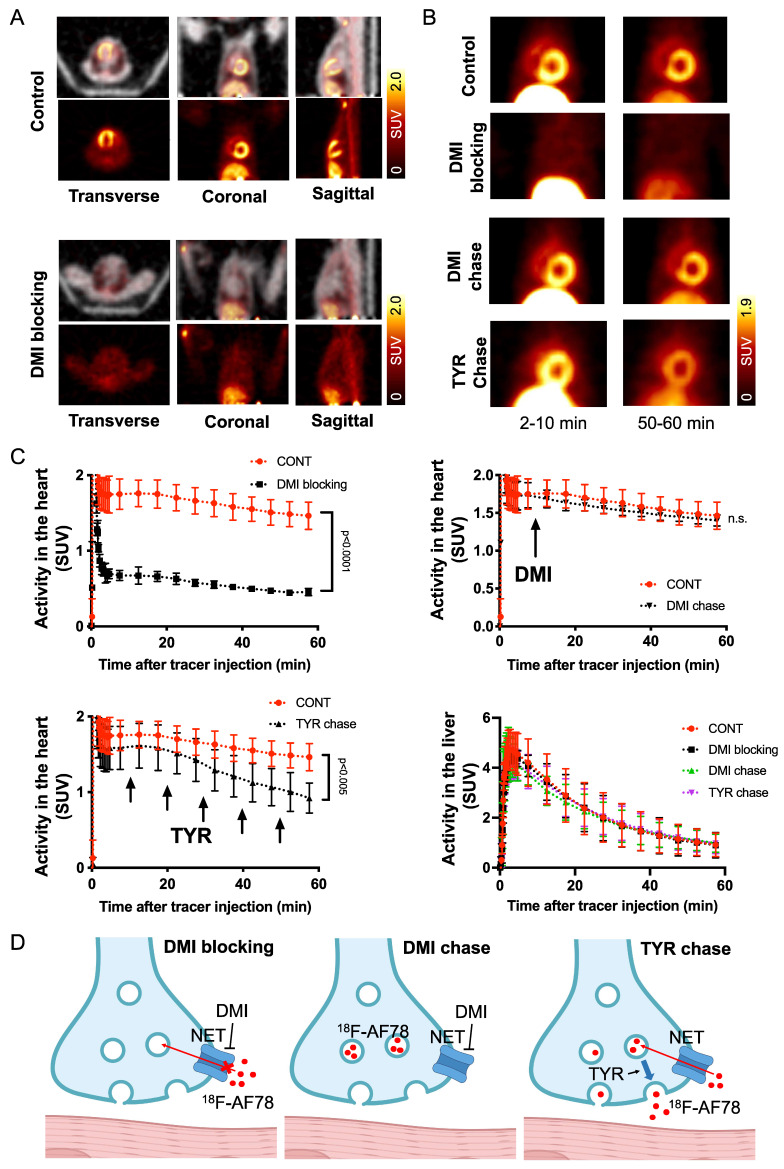
**(A)** PET/CT fused images (top row of each image) of ^18^F-AF78 in control nonhuman primates (NHPs) together with inhibition of norepinephrine transporter (NET) by using desipramine (DMI). Images were acquired 40-60 min after tracer injection. Distinct cardiac uptake of ^18^F-AF78 was visualized in control animals. In animals pretreated with the selective NET blocker DMI (10 min before the tracer injection), cardiac uptake diminished, supporting the notion of high affinity to the NET across species. **(B)** PET images of ^18^F-AF78 in NHPs at both early (2-10 min after tracer injection) and late stages (50-60 min after tracer injection) in control, DMI pretreatment, DMI chase and tyramine (TYR) chase conditions. **(C)** Kinetic studies of ^18^F-AF78 in NHPs with DMI blocking (top left), DMI chase (top right), tyramine (TYR) chase (bottom left) together with activity in the liver (bottom right) illustrated as time-activity curves (standardized uptake value (SUV) *versus* time in minutes, *n* = 4) in the heart. The cardiac uptake of ^18^F-AF78 was specifically blocked by pretreatment with the NET blocker DMI (10 min before tracer i.v.), but was not sensitive to a single-time DMI chase protocol (DMI administration 10 min after tracer i.v.). The intermittent TYR chase protocol (tracer i.v. followed by injection of TYR 5 times in a 10-min interval) markedly decreased the radiotracer retention in the myocardium. Activity in the liver remained consistent in different conditions, demonstrating the NET specificity of ^18^F-AF78 and independent from pharmacological conditions. *n* = 4 animals were investigated. Please refer to the supplementary material for the experimental timeline and detailed protocol (Fig. S1). **(D)** Illustrated mechanisms of kinetic studies for clarification of the results.

**The kinetic profile of ^18^F-AF78 demonstrated great application potential for cardiac SNS imaging.** DMI-mediated selective NET inhibition led to markedly reduced radiotracer accumulation in NHP hearts relative to controls, demonstrating high specificity (**Figure 5A**). The cardiac uptake of ^18^F-AF78 was retained at 60 min after radiotracer administration, which could not be diminished by DMI chase but by TYR chase (**Figure 5B**). Time-activity curves demonstrated a significant decrease and could not clearly identify the delineation of left ventricular radiotracer uptake after pretreatment with the NET selective blocker DMI (mean SUV at 60 min, DMI blocking 0.45 ± 0.05 vs. controls, 1.46 ± 0.18;* P* ≤ 0.0001;** Figure 5C**). In contrast, cardiac washout was not affected by a single-time chase protocol using the NET inhibitor DMI after the administration of ^18^F-AF78, suggesting stable radiotracer retention independent of NET activity after initial transport (mean SUV at 60 min, DMI chase and controls, 1.40 ± 0.07 and 1.46 ± 0.18, respectively; n.s.; **Figure 5C**). Intermittent chasing with the catecholamine stimulant TYR at a 10-min interval, however, markedly reduced cardiac tracer retention (mean SUV at 60 min, 0.92 ± 0.20, vs. 1.46 ± 0.18; *P* ≤ 0.005;** Figure 5C**), supporting the notion that ^18^F-AF78 is stored in synaptic vesicles, while its spillover increases in response to TYR stimulation. Hepatic uptake and washout kinetics, however, were not affected in all different conditions of treatment and remained consistent (**Figure 5C**), indicating the high specificity of ^18^F-AF78 for NET.

## Discussion

Here, we focused on the NET as the first important step of radiotracer handling at the presynaptic nerve terminal in the heart and systemically evaluated the recently introduced PET agent ^18^F-AF78 in a cell-based competitive uptake assay. Additionally, we compared its NET affinity to the reference standard NE, MIBG, HED, and other established ^18^F-labeled SNS radiotracers. In a human neuroblastoma cell line naturally expressing NET, ^18^F-AF78 demonstrated an IC_50_ value comparable to that of physiological NE. Among all investigated radiotracers, AF78 shows potential as a fluorine-18-labeled PET ligand for SNS imaging with an affinity comparable to that of HED but significantly more potent than MIBG (**Table 1**). These results serve as an encouraging complement to a previous DMI blocking study, in which NET specificity was similar to that of ^131^I-MIBG; reverse blocking using either ^18^F-AF78 or ^131^I-MIBG has already demonstrated an encouraging *in vitro* profile 20. Moreover, *in vivo* studies revealed a favorable biodistribution with good heart-to-blood pool, heart-to-lung and heart-to-liver ratios in various animal species, thereby allowing for clear contrast imaging of the LV, especially at late time points demonstrated in both rats and NHPs. In addition, ^18^F-AF78 uptake was sensitive to NET-selective inhibition prior to radiotracer injection, indicating a high specificity to neuronal NET in both rabbits and NHPs. Furthermore, unlike HED undergoing continuous cycling (i.e., diffusion and reuptake) at the nerve terminal, cardiac SNS uptake of ^18^F-AF78 in NHPs was resistant to DMI chase, suggesting stable radiotracer retention independent of NET activity after initial transport. Last, intermittent chase with the NE stimulator TYR led to a marked spillover of ^18^F-AF78 from the heart, supporting the notion of stable storage and release kinetics similar to its physiological reference, NE. Overall, the kinetic profile can be used to reflect the mechanistic integrity of NE vesicular turnover pathways, particularly at the presynaptic nerve terminal (**Figure 5C, D**) 26, rendering ^18^F-AF78 a highly promising SNS imaging agent. These encouraging results should trigger further studies of ^18^F-AF78, including assessment of biodistribution in humans or to test its clinical significance in multiple challenging scenarios, such as ischemic and nonischemic HF, diabetes-related CAN, valvular heart diseases, cardiomyopathies, or cardiac arrhythmias, including the guidance of resynchronization therapies 8.

In light of these encouraging results, the presented findings should trigger further head-to-head comparisons for quantitative assessments of denervated myocardium between ^18^F-AF78 and other reported radiotracers. These considerations are further fueled by the fact that, in contrast to the relatively constant hepatic uptake of ^11^C-HED, ^18^F-AF78 demonstrated fast liver washout in NHPs (**Figures 4B, 5C**) in a manner similar to other ^18^F-labeled radiotracers, as demonstrated by the increasing heart-to-liver ratios over time (5 min after tracer i.v., 0.38 ± 0.29 vs. 30 min, 0.95 ± 0.20 vs. 60 min, 1.60 ± 0.38) 27, 28. In comparison, the heart-to-liver ratios of ^18^F-4F-MHPG and ^18^F-3F-PHPG measured in rhesus macaque monkeys reached 2.2 ± 0.8 and 2.5 ± 0.3, respectively, not before 90 min after radiotracer injection 27. In contrast, cardiac retention of ^18^F-AF78 decreased only slightly in the time-activity curves, in which the SUV dropped from 1.74 ± 0.24 at 5 min to 1.46 ± 0.18 at 60 min postinjection (**Figure 5C**). A similar pattern of fast liver washout has also been observed with ^18^F-3F-PHPG and ^18^F-4F-MHPG or ^18^F-FBBG in human studies 23, 29. Therefore, clinical protocols should carefully address the issue of high hepatic uptake at early time points, e.g., by employing an appropriate waiting time after tracer injection before scan initiation. In addition, different hepatic time-activity curves seen in rats and NHPs could also be explained by plasma metabolism due to species discrepancy, and future studies are required to investigate such metabolic aspects. Considering the monoamine oxidase-resistant guanidine tracer structure and relatively stable ether bond, the passive diffusion of the tracers from storage vesicles due to increased lipophilicity through the introduction of a 3-fluoropropyl moiety could explain the higher clogP compared to the lead structure 3F-PHPG 1.27 vs. AF78 1.83, and lower value compared to MIBG 2.72 (clogP values calculated with MarvinSketch 20.13.0, ChemAxon). In the heart, secretory vesicles are acidic under physiological conditions and maintain a pH of 5.0-5.7, depending on the conditions 30, while radiotracers with guanidine moieties are more basic than physiological neurotransmitters with higher pKa values (NE 9.74, MIBG 11.75, MHPG 12.3, FBBG 11.68 and AF78 12.08 (pKa values calculated with MarvinSketch 20.13.0, ChemAxon)). Therefore, on the one hand, after active transport into vesicles, they are stably stored in the heart; on the other hand, the liver uptake and washout rate may at least partially relate to their lipophilicity/clogP value. Further cellular studies are necessary, especially to investigate the Vmax and km values of different tracers to establish kinetics models and clarify the structure-activity relationships of NET-targeting radiotracers.

Although all cardiac sympathetic innervation radiotracers are transported to the nerve terminals in the heart, their subcellular kinetics vary in terms of intracellular storage mode, exemplified by ^11^C-HED 22. Compared to the stable retention of ^123^I-MIBG or ^18^F-FBBG in storage vesicles, ^11^C-HED undergoes continuous diffusion and NET-mediated reuptake to maintain equilibrium, as shown by decreased cardiac radiotracer uptake after the DMI chase 22. On the one hand, ^18^F-AF78 demonstrated stable radiotracer retention in the nerve terminals, resistant to the DMI chase (**Figure 5D**). However, TYR led to the release of ^18^F-AF78, likely via two different mechanisms 31. First, by releasing the storage vesicles at the nerve terminals, and second, by acting as a substrate to the NET, the initial transport of TYR into the neuron initiates reverse transport of NE and radiolabeled neurotransmitters within the cytoplasm 32. Thus, the releasing effect was initiated after the intermittent administration of TYR (**Figure 5C**); thereafter, an equilibrium would be reached over time since the released radiotracer would be transported back into the nerve terminal, likely due to the high specificity of ^18^F-AF78 to NET, in a pattern similar to that of ^18^F-FBBG 33.

**Figure 6:**
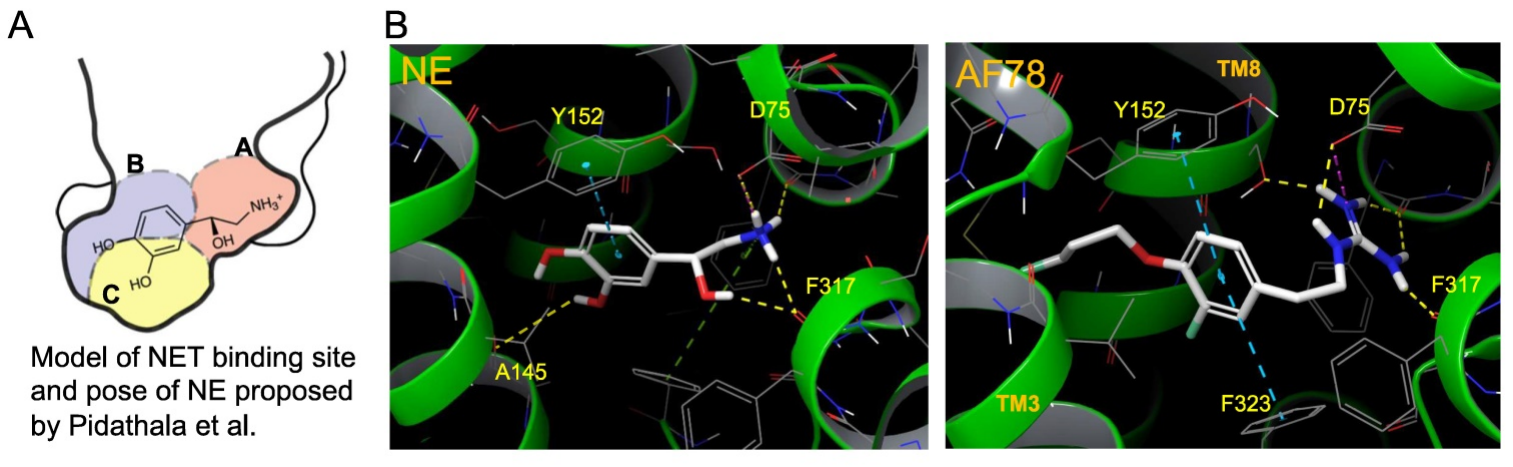
Model of the NET-binding site and proposed binding poses of molecules in a docking study to rationalize the high affinity of AF78. **(A)** Proposed model of the NET-binding site using the *Drosophila melanogaster* dopamine transporter as a surrogate of NET 33. **(B)** Docking of NE (left) and AF78 (right) into the 3D structure of NET adopting the conformation of PDB6M0Z followed by *AlphaFold* prediction and *induced fit docking*. White sticks represent the carbon backbones of the molecules, red represents oxygen, blue represents nitrogen, and green represents fluorine. Helixes of NET, such as TM3 and TM8, are green. The formation of a stacked T-shaped π-π interaction between the benzene ring of AF78 and amino acid moieties Y152 and F323 enhanced by *meta*-fluoride is crucial for its high NET affinity. A lack of hydrogen bonds, similar to NE, is compromised by this π-π stacking and stabilized by *meta*-fluoride.

In addition to the *in vitro* and *in vivo* studies, an *in silico* hypothesis might hint at a potential explanation for the high NET affinity of AF78. Because there is currently no available X-ray structure of NET, PDB6M0Z, a dopamine transporter with NET-like mutations (D121G/S426 M/F471 L) in NE-bound form, was first chosen for the docking study 34. Adopting the conformation of PBD6M0Z, the 3-dimensional structure of NET as a Homo-AF model was established using AlphaFold prediction. In this model, the smaller radiotracers, including HED, MIBG, 4F-MHPG, 3F-PHPG and 6F-DA (**Table 1**), can be docked into the primary binding site in a pattern similar to NE, with amino or guanidine groups that fit into the binding pocket A, while those radiotracers with a hydroxy group may form a hydrogen bond with A145 according to the model established by Pidathala (**Figure 6**) 34. However, the molecules with a “tail” (3-fluoropropyl group, e.g., FBBG and AF78) do not fit well into the proposed binding pockets as the “tail” flips between binding pocket B and C (**Figure 6A**). Therefore, induced-fit docking was performed to allow a better and higher range of rotation of the amino acid moieties. After this adjustment, only one AF78 orientation was present, with the 3-fluoropropyl group pointing to a hydrophobic binding pocket between TM3 and TM8, in accordance with pocket B in Pidathala's model. Most importantly, in this binding position, the fluoride points to pocket C, and the benzene ring of AF78 forms a stacked T-shaped π-π interaction with Y152 and F323, i.e., the benzene ring of AF78 is perpendicular to both amino acid moieties (**Figure 6B**). Due to the strong electronegativity of the *meta*-fluorine substituent, this stacked π-π interaction may be enhanced, thereby stabilizing the binding of AF78 to NET, although AF78 lacks an important hydroxy group similar to other radiotracers that are responsible for a hydrogen bond. The T-shaped π-π stacking configuration is positively correlated with the electronegativity of the *meta*-substitution on the benzene ring, i.e., F<Cl<Br<I<OH. From both electrostatic effect and exchange-repulsion terms, strong electron-withdrawing fluoride is favorable, which decreases the electron density of the benzene ring of AF78(F), while forming partially positively charged hydrogen that points at the negatively charged π cloud of the benzene ring above the tyrosine moiety 35.

Overall, the resistance to DMI chase together with the susceptibility to intermittent TYR chase indicates the mode of transportation and storage of ^18^F-AF78 in sympathetic neurons, which is characterized by active transport via NET followed by stable storage in presynaptic vesicles. Of note, such an *in vivo* kinetic profile represents a similar pattern as ^123^I-MIBG and ^18^F-FBBG 22. In addition, it is especially worth mentioning that the pilot imaging studies in patients with heart failure using ^18^F-3F-PHPG and ^18^F-4F-MHPG, which share the phenethylguanidine core structure with AF78, have provided reliable quantitative metrics of regional sympathetic nerve density 36. As a result, given its high affinity to the subcellular target and high-contrast images in various animal species,^ 18^F-AF78, as a second-generation SNS radiotracer that also has a phenethylguanidine core structure, may be readily applicable for human use.

This study has several limitations. Future studies should also focus on the ^18^F-AF78 interactions in the presynaptic nerve terminal, e.g., by inhibiting the vesicular monoamine transporter 2 (VMAT2) using tetrabenazine. Such studies are important as NE analogs are subject to vesicular packaging by this transporter 37. Another factor affecting uptake and retention is the specific activity of the radiotracer, as has been demonstrated by our research on ^11^C-HED in rat hearts. 38. The cold mass effect should certainly be addressed after establishing a fully repeatable radiolabeling protocol suitable for clinical application. The interpretation of the tyramine induced catechol release should be carefully considered, because it may interact with several targets , such as VMAT2, monoamine oxidase, and alpha receptors. Therefore, further studies using reserpine may provide more solid support for the submolecular storage and release mechanism of AF78. Moreover, given the low number of investigated animals per group due to ethical issues, statistical analyses are limited, and a weak effect could have been missed. Increased neuronal retention times and lower neuronal uptake rates have been advocated to provide more reliable read-outs of nerve integrity, as the blood flow dependency is minimized, while the NET transport rate would be slower 39. Therefore, future studies should also investigate these parameters of ^18^F-AF78 relative to established nerve radiotracers, preferably in dedicated disease models at late time points 90-120 min postinjection, e.g., in heart failure following myocardial infarction. Nonetheless, a comprehensive evaluation of the radiotracer kinetics in healthy animals, as presented in the present study, may be valuable as it allows us to adequately interpret findings in preclinical scenarios with deteriorating cardiac performance. As such, the present study mapping the interactions of ^18^F-AF78 at the NET in various (healthy) species may be interpreted as laying the proper groundwork for important follow-up studies. Further studies will also provide information on tracer uptake related to sympathetic nerve density or NE content 40. Moreover, test-retest studies evaluating the reproducibility of radiotracer kinetics in various species and humans are needed to ensure that the interpreting molecular imaging expert has high certainty regarding the robustness of the derived cardiac nerve integrity information.

## Conclusion

Relative to the physiological reference NE, the second-generation NET radiotracer ^18^F-AF78 demonstrated almost identical *in vitro* affinity to NET and was comparable to other established ^18^F-labeled SNS radiotracers and MIBG. Studies across various species demonstrated reassuring biodistribution and a positive kinetic profile, including specific NET-mediated transport, followed by a stable storage and release mode. Computational modeling also provided a rationale for its high NET affinity. These results render ^18^F-AF78 a promising SNS radiotracer for prognostication and molecular image-guided strategies in future cardiovascular medicine, e.g., to guide interventions such as resynchronization therapies in ischemic and nonischemic HF, diabetes-related CAN or valvular heart diseases. Beyond cardiovascular diseases, further applications include systems-based organ analyses by providing holistic benefit for the target (heart) and remote organ (kidney) in patients with cardiorenal syndrome or in a cardio-oncology setting, e.g., to prevent relevant cardiotoxicity of anticancer therapeutics.

## Supplementary Material

Supplementary figures.

## Figures and Tables

**Table 1:** Competitive cell uptake affinity against ^3^H-norepinephrine (^3^H-NE) in human neuroblastoma cells.

Testing Compound ^#^	Structure ^†^	IC_50_ (µM)^ ‡, §^
DMI	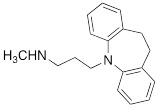	0.010 ± 0.0015^*^
PhB	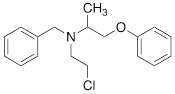	0.82 ± 0.30^ n.s.^
NE	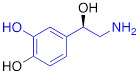	0.50 ± 0.16
6F-DA	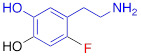	1.20 ± 0.33^*^
MIBG	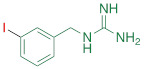	1.75 ± 0.47^**^
FBBG	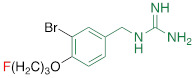	2.28 ± 1.05^*^
4F-MHPG	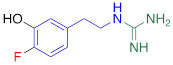	2.78 ± 1.22^**^
3F-PHPG	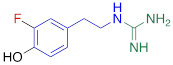	0.78 ± 0.060^ n.s.^
HED	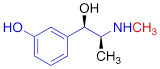	0.39 ± 0.11^ n.s.^
AF78	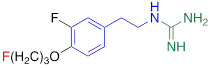	0.42 ± 0.14^ n.s.^

**^#^**NE = norepinephrine DMI = desipramine. PhB = phenoxybenzamine. 6F-DA = 6-fluorodopamine. FBBG = flubrobenguane. 4F-MHPG = 4-fluoro-3-hydroxyphenethylguanidine. 3F-PHPG = 3-fluoro-4-hydroxyphenethylguanidine. HED = metahydroxyephedrine. MIBG = metaiodobenzylguanidine. AF78 = 1-(3-fluoro-4-(3-fluoropropoxy)phenethyl)guanidine. **^†^**In addition to NET blockers DMI and PhB, the radiotracers are colored to show the similarity in their chemical structures. Red represents a radionuclide acting as a radiotracer. **^‡^**Values are presented as the means ± SD for individual assays (*n* = 4). **^§^**Compared to NE, where ^n.s.^ *P* > 0.05, ^*^ *P* ≤ 0.05, ^**^ *P* ≤ 0.01.

## Abbreviations

6F-DA: 6-fluorodopamine
A.U.: arbitrary unit
CAN: cardiac autonomic neuropathy
DMI: desipramine
HED: *meta*-hydroxyephedrine
HF: heart failure
ICD: implantable cardiac device
i.v.: intravenous
FBBG: flubrobenguane
MIBG: *meta*-iodobenzylguanidine
NE: norepinephrine
NET: norepinephrine transporter
NHP: nonhuman primate
PD: Parkinson's disease
PET: positron emission tomography
SNS: sympathetic nervous system
SPECT: single-photon emission computed tomography
SUV: standardized uptake values
TYR: tyramine
