# Supplementary material for: Molecular Imaging-Derived Biomarker of Cardiac Nerve Integrity — Introducing High NET Affinity PET Probe 18F-AF78
Source: Theranostics. 2022 May 24;12(9):4446–58. doi: 10.7150/thno.63205 (PMC9169360; doi:10.7150/thno.63205)
Supplement: Supplementary file 1 — Supplementary figures. [file thnov12p4446s1.pdf]

Supplementary Material

of

A Molecular Imaging-Derived Biomarker of Cardiac Nerve Integrity —

Introducing High NET Affinity PET Probe ^{18}F-AF78

Xinyu Chen*[a,b], Rudolf A. Werner*[b,c], Kazuhiro Koshino[d], Naoko Nose[e], Saskia Mühlig[b], Steven P. Rowe, PhD[c], Martin G. Pomper[c], Constantin Lapa[a], Michael Decker[f], Takahiro Higuchi[b,e]

[a]*Nuclear Medicine, Faculty of Medicine, University of Augsburg, Augsburg, Germany*
[b]*Department of Nuclear Medicine and Comprehensive Heart Failure Center, University Hospital of Würzburg, Würzburg, Germany*
[c]*Division of Nuclear Medicine, The Russell H Morgan Department of Radiology and Radiological Science, Johns Hopkins University School of Medicine, Baltimore, MD, U.S.*
[d]*Department of Systems and Informatics, Hokkaido Information University, Ebetsu, Hokkaido, Japan*
[e]*Graduate School of Medicine, Dentistry and Pharmaceutical Sciences, Okayama University, Okayama, Japan*
[f]*Institute of Pharmacy and Food Chemistry, University of Würzburg, Würzburg, Germany*
*equal contribution

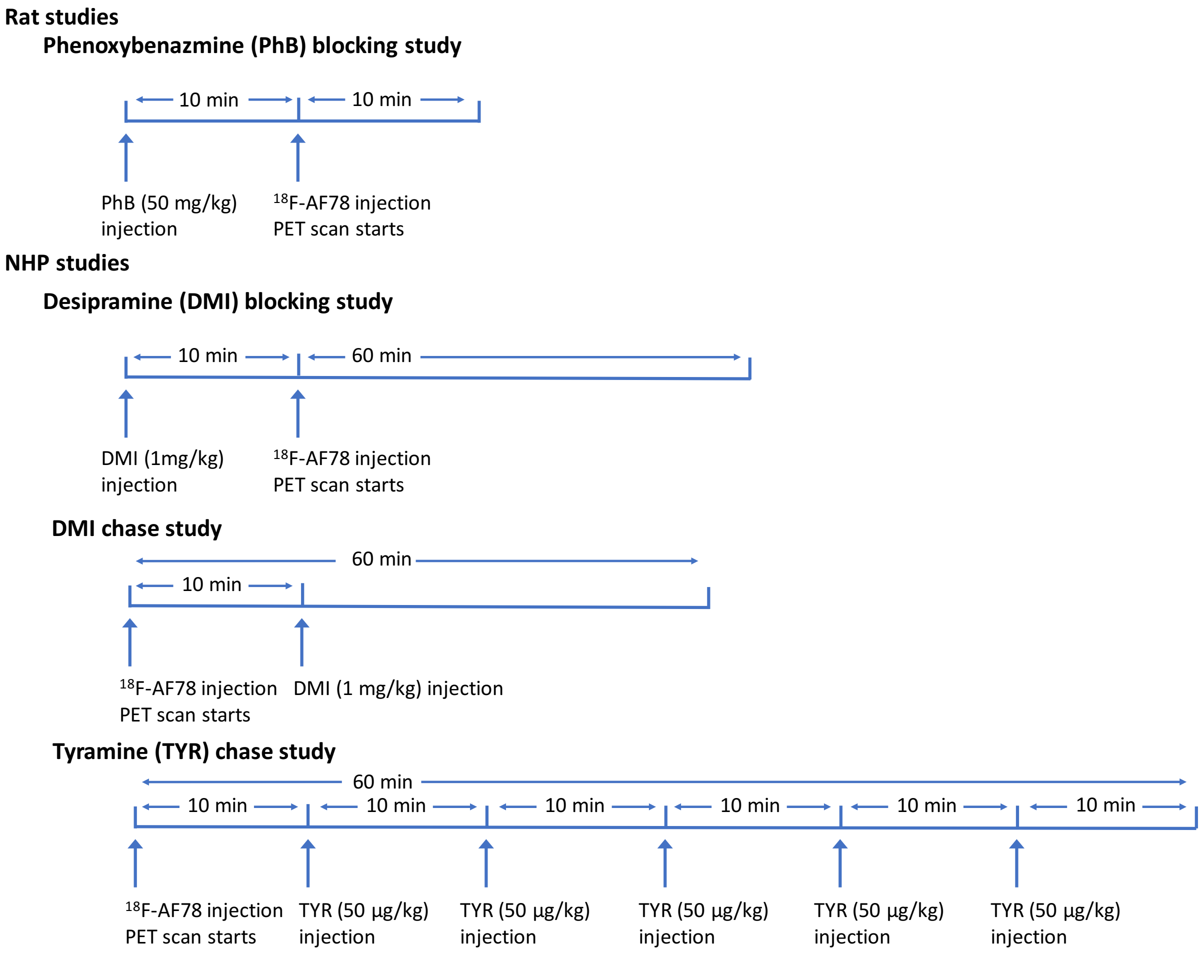

Figure S1. Experimental timeline of rat and non-human primates (NHPs) studies. PET = positron emission tomography.

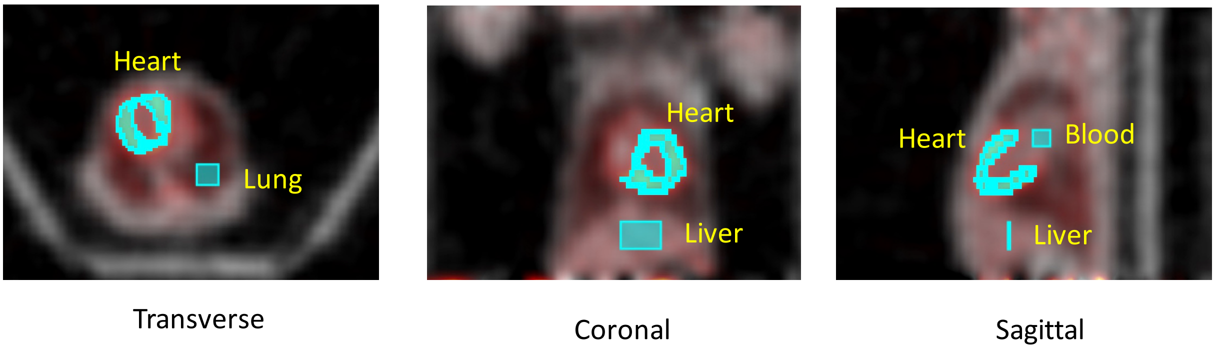

Figure S2. Region of interest placement, exemplified by the positron emission tomography/transmission fused image of ^{18}F-AF78 in control non-human primates. Heart, blood pool, liver and lung have been indicated.
